# Ivermectin resistance mechanisms in ectoparasites: a scoping review

**DOI:** 10.1007/s00436-024-08223-z

**Published:** 2024-05-24

**Authors:** Joanna Furnival-Adams, Caroline Kiuru, André Barembaye Sagna, Karine Mouline, Marta Maia, Carlos Chaccour

**Affiliations:** 1 Barcelona Institute for Global Health (ISGlobal), Barcelona, Spain; 2 Facultat de Medicina i Ciències de la Salut, Universitat de Barcelona (UB), Barcelona, Spain; 3 Centro de Investigação Em Saúde de Manhiça (CISM), Maputo, Mozambique; 4 MIVEGEC, University of Montpellier, IRD, CNRS, Montpellier, France; 5 Kenya Medical Research Institute, Wellcome Trust Research Programme, Kilifi, Kenya; 6 Centre for Tropical Medicine and Global Health, Nuffield Department of Medicine, University of Oxford, Oxford, UK; 7 CIBER de Enfermedades Infecciosas, Madrid, Spain; 8 Universidad de Navarra, Pamplona, Spain

**Keywords:** Ivermectin, Insecticide resistance, Endectocide, Mosquitoes, Ectoparasites

## Abstract

Ivermectin mass drug administration has been used for decades to target human and veterinary ectoparasites, and is currently being considered for use against malaria vectors. Although there have been few reports of resistance to date in human ectoparasites, we must anticipate the development of resistance in mosquitoes in the future. Hence, through this review, we mapped the existing evidence on ivermectin resistance mechanisms in human ectoparasites. A search was conducted on the 8th November 2023 through databases, PubMed, Web of Science, and Google Scholar, using terms related to ivermectin, human and veterinary ectoparasites, and resistance. Abstracts (5893) were screened by JFA and CK. Data on the study organism, the type of resistance, the analysis methods, and, where applicable, the gene loci of interest were extracted from the studies. Details of the methodology and results of each study were summarised narratively and in a table. Eighteen studies were identified describing ivermectin resistance in ectoparasites. Two studies described target site resistance; and 16 studies reported metabolic resistance and/or changes in efflux pump expression. The studies investigated genetic mutations in resistant organisms, detoxification, and efflux pump expression in resistant versus susceptible organisms, and the effect of synergists on mortality or detoxification enzyme/efflux pump transcription. To date, very few studies have been conducted examining the mechanisms of ivermectin resistance in ectoparasites, with only two on *Anopheles* spp. Of the existing studies, most examined detoxification and efflux pump gene expression, and only two studies in lice investigated target-site resistance. Further research in this field should be encouraged, to allow for close monitoring in ivermectin MDA programmes, and the development of resistance mitigation strategies.

**Graphical Abstract:**

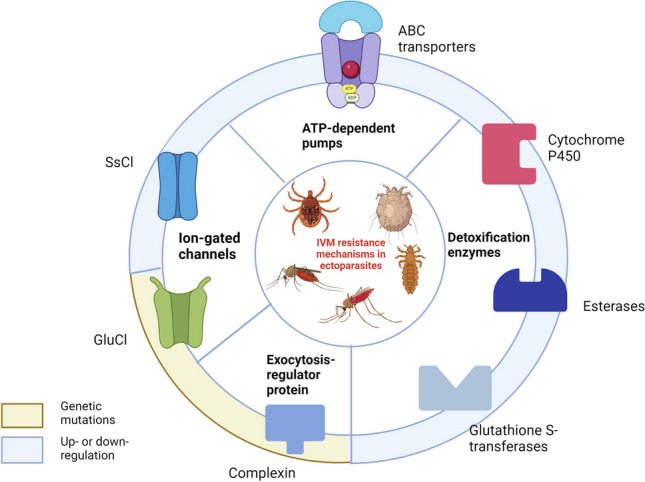

**Supplementary Information:**

The online version contains supplementary material available at 10.1007/s00436-024-08223-z.

## Background

Ivermectin is widely used in the treatment of veterinary and human endoparasites and ectoparasites, and is now being considered for use in mass drug administration (MDA) against vectors of malaria (Billingsley et al. 2020). In this context, ivermectin is administered to humans and/or livestock, and susceptible host-seeking mosquitoes that feed upon treated subjects with sufficient concentrations of ivermectin will die within a few days thereby reducing malaria transmission rates (Chaccour et al. 2010). The plasma half-life of ivermectin varies depending on the dose, formulation, and regimen; however, studies suggest that it is between 1 and 3 days for a single dose of 150–200 μg/kg (STROMECTOL 1996; Duthaler et al. 2019). Despite this relatively short half-life, there are reports of increased mortality in mosquitoes feeding on the treated host up to 28 days after the administration of ivermectin when 300 μg/kg a day 3 days in a row is given, which may be due to ivermectin metabolites that continue to circulate post-ivermectin clearance (Smit et al. 2018; Kern et al. 2023). While these data refer to oral dosing of humans, new, long-lasting ivermectin formulations currently under development will have different pharmacokinetic and mosquito-killing profiles (Pooda et al. 2023; Lyndra Inc MAMBJ 2016).

The use of any mono-therapeutic drug in MDA as a disease control method provokes concern regarding its potential to induce drug resistance selection, particularly if the parent compound or its metabolites have a long elimination tail (Smits 2014). If mosquito populations with heterogeneous traits are systematically exposed to sublethal levels of ivermectin, this may result in the selection of genes or genetic loci in the population that increase their chances of surviving exposure to ivermectin. In this review, we map and discuss the mechanisms of ivermectin resistance seen in ectoparasites of human importance. We consider the term “ectoparasites” to include all biting, blood-feeding arthropods, including insects and arachnids.

### Reports of resistance to ivermectin

In several veterinary parasite populations, practical resistance to ivermectin appeared shortly after it became available and is now widespread (Shoop 1993; Laing et al. 2017). Resistance in roundworms of sheep, horses, and cattle is now a significant concern in the veterinary world, and resistance has also been reported in other helminths such as the canine heartworm (Kaplan 2004). There have also been numerous reports of both practical and technical ivermectin resistance in veterinary ectoparasites, predominantly in ticks (Singh et al. 2015; El-Ashram et al. 2024; Fernández-Salas et al. 2012). In human ectoparasites, there have been sporadic reports of practical resistance, for example in scabies mites and headlice following extensive treatment with ivermectin (Currie et al. 2004; Amanzougaghene et al. 2018b). However, despite widespread use of ivermectin over the past few decades for several indications, reports of resistance to date have been limited and contentious (Crump et al. 2011). Two studies have suggested suboptimal responses in individuals with onchocerciasis that had received yearly doses of ivermectin over the course of several years (Osei-Atweneboana et al. 2011; Awadzi et al. 2004). In both cases, however, it is not clear whether the non-responsiveness was due to resistance or due to other operational factors. Regardless of the scarcity of ivermectin resistance reports in human ectoparasites to date, and given experience from other insecticides and antibiotics, we must anticipate the development of resistance in *Anopheles* mosquitoes and make projections about how and when it will develop.

In addition to resistance in mosquitoes, the scale-up of ivermectin for use in malaria control may induce effects on other ectoparasites for which ivermectin is indicated, such as scabies mites, headlice, and ticks. Resistance in these organisms should also be monitored and strategically delayed, where possible.

### Possible ivermectin resistance mechanisms

In nematodes, extensive research has indicated that common mechanisms of resistance include GluCl mutations, changes to ABC transporter expression, and through upregulation of detoxification genes (Dent et al. 2000; James and Davey 2009; Cile Mé Nezid et al. 2019). However, the mechanisms of ectoparasitic resistance are less clear. In arthropods in general, target-site resistance is a common mechanism of insecticide resistance. In ectoparasites and arthropods such as *Drosophila melanogaster*, ivermectin is known to target ligand-gated chloride channels, primarily glutamate-gated ion channels (Laing et al. 2017; Martin et al. 2020). This has also been demonstrated in *Anopheles* and *Culicine* mosquitoes (Meyers et al. 2015). Studies have suggested that other ion channels, such as histamine-gated chloride channels, pH-gated chloride channels, nicotinic acetylcholine receptors (nAChRs), and gamma amino butyric acid (GABA)-gated chloride channels are also targeted, but this may be a downstream effect as a result of glutamate-gated chloride (GluCl) channel inhibition (Fuse et al. 2016; Atif et al. 2019). Although there is relative uncertainty on the ivermectin targets in mosquitoes, it is possible that mutations in the genes coding for these channels will result in insecticide resistance.

Metabolic resistance is also a common form of resistance, where increased enzymatic degradation of insecticidal molecules occurs via for instance, increased expression of genes involved in detoxification mechanisms such as cytochrome P450 (Feyereisen et al. 2015). This is often observed in mosquito resistance to other insecticides, and detoxification occurs before molecules reach the target-site (Balabanidou et al. 2016). Strategies have been used to counteract this by incorporating synergists that inhibit cytochrome P450 such as Piperonyl-butoxide (PBO) into existing interventions (Protopopoff et al. 2018). Finally, changes in efflux pump expression are associated with multi-resistant mosquitoes. Their role in resistance may be linked to the protection of target sites in the nervous system and/or accelerated clearance of insecticides (Pignatelli et al. 2018).

Using existing evidence, we summarise mechanisms of physiological ivermectin resistance that are known to exist in ectoparasites to understand which mechanisms may arise in mosquitoes. Predicting mechanisms of resistance will facilitate the development of molecular assays, enable preparedness of monitoring and evaluation strategies, and the development of plans to mitigate the effect of resistance.

### Research question

What is known in the existing literature about ivermectin resistance in ectoparasites of human importance and their biochemical/molecular mechanisms?

## Methods

### Search databases

We conducted a literature search using electronic databases (PubMed, Web of Science and Google Scholar) on the 8th November 2023 using search terms that include concepts related to ivermectin, resistance, and any of the organisms mentioned in the inclusion criteria (Table 1). Two authors (JFA and CK) screened abstracts independently using Rayyan. There were no restrictions with respect to date or language. The reference lists of each included study were also used to identify any additional studies.

**Table 1:** Concepts and search terms used for each respective database

Database	Concept	Search terms
PubMed	Resistance	resistance [tw] OR "insecticide resistance" [tw] OR mutation [tw] OR "suboptimal response*" [tw] OR resistant [tw] OR "Insecticide Resistance"[Mesh]
	Ectoparasites	mosquito* [tw] OR Aedes [tw] OR Anopheles [tw] OR Culex [tw] OR scabies [tw] OR sarcoptes [tw] OR scabiei [tw] OR headlice [tw] OR headlouse [tw] OR pediculosis [tw] OR cimex [tw] OR hemipterus [tw] OR lectularius [tw] OR bedbug* [tw] OR tick* [tw] OR"culicidae"[MeSH Terms] OR "scabies"[MeSH Terms] OR "lice infestations"[MeSH Terms] OR "Sarcoptes scabiei"[Mesh] OR "Bedbugs"[Mesh] OR "Ticks"[Mesh]
	Ivermectin	"macrocyclic lactone*" OR ivermectin OR abamect* OR avermect* OR "ivermectin"[MeSH Terms]
Google Scholar	Resistance	(resistance OR "insecticide resistance" OR mutation OR "suboptimal response*" OR resistant)
	Ectoparasites	(mosquito OR Aedes OR Anopheles OR Culex OR scabies OR sarcoptes OR scabiei OR headlice OR headlouse OR pediculosis OR cimex OR hemipterus OR lectularius OR bedbug* OR tick* OR "culicidae" OR "scabies" OR "lice infestations" OR "Sarcoptes scabiei" OR "Bedbugs" OR "Ticks")
	Ivermectin	("macrocyclic lactone*" OR ivermectin OR abamect* OR avermect* OR "ivermectin")

### Inclusion criteria

We included studies investigating resistance mechanisms in human or veterinary ectoparasites including scabies mites, ticks, bed bugs, headlice and body lice, or mosquitoes. Studies that have described (a) genetic mutations, (b) biochemical changes that have emerged through selection pressure either in vitro or in vivo, and (c) synergist assays investigating the detoxification of ivermectin were included. We excluded reviews lacking primary data, reports of resistance to macrocyclic lactones that are not part of the avermectin family, or reports of resistance to ivermectin in non-biting arthropods such as *Drosophila*, spider mites, or diamondback moths.

### Data extraction and management

Data were extracted independently by JFA, CK, and AS. The following data were extracted from each article:

- Organism
- Origin of sample (including whether the study was in vivo or in vitro)
- Sample size
- Study design
- Resistance status of study organism (suspected resistance, confirmed resistance, confirmed reduced tolerance, susceptible)
- Mechanism of resistance (target-site/metabolic/efflux pump-related, gene location, corresponding mutation, or metabolite implicated in the resistance mechanism)
- Intensity of resistance

The data were summarised in a table, and the methodology, setting, and results of each study were synthesised narratively. This scoping review was reported according to the Preferred Reporting Items for Systematic Reviews and Meta-Analyses extension for Scoping Reviews (PRISMA-ScR) (Additional File 1) (Tricco et al. 2018).

### Deviations from the original protocol

After our initial search, we identified several studies on sea lice that met the inclusion criteria. However, after a joint discussion, we considered the biology of these organisms to be notably different from those relevant to the research question. Therefore, we decided post hoc to restrict the inclusion of studies on veterinary ectoparasites, to only those that are capable of infesting or feeding on human hosts.

## Results

### Characteristics of included studies

**Fig. 1:**
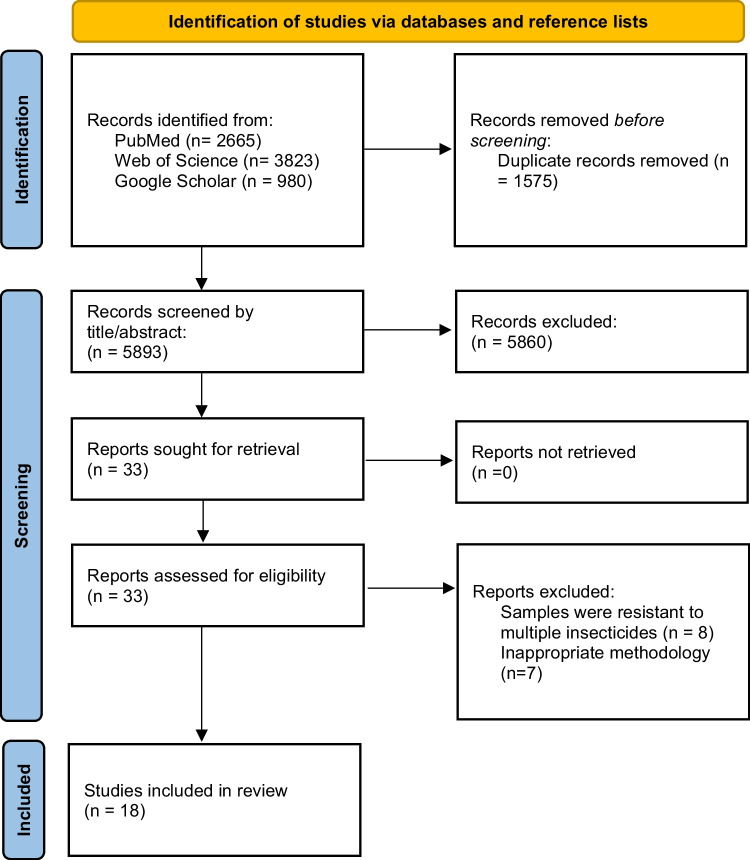
PRISMA flow chart

The search generated 5893 unique articles or abstracts published before 8th November 2023 (Fig. 1). 33 full-texts were screened, and 18 articles met the inclusion criteria. The studies investigated resistance mechanisms in lice (*n* = 4), ticks (*n* = 9), mites (*n* = 2), and mosquitoes (*n* = 3) (Table 2). Measurements used to assess the presence (and in some cases, the strength) of resistance included reports from doctors and their patients experiencing clinical treatment failure (*n* = 3), measurements of resistance ratio (RR50 or RR90) based on lethal times (*n* = 2), or studies where resistance measures where reported as a resistance factor (RF) (*n* = 6), and cell survival rates (*n* = 1). Based on these measurements, the samples from these studies were all classified as resistant, aside from one, which was determined to have decreased tolerance to ivermectin. Six studies were conducted in susceptible organisms. Studies investigated mechanisms of target-site resistance (*n* = 2), or metabolic resistance (*n* = 10), and/or changes to multi-resistant protein (MRP) transporter expression (*n* = 12). Methodologies included genetic sequencing (*n* = 1), transcriptional profiling (*n* = 8) of selected gene loci, or synergist bioassays (*n* = 9) (using synergists known to inhibit genes expression associated with detoxification mechanisms).

**Table 2:** Summary of the methods and resistance mechanisms investigated in the studies identified

Study ID	Species	Host organism	Resistance generation*			Resistance status**	Resistance type			Type of analysis	Gene loci
			FC	LR	S		Target-site	Metabolic	MRP*** Transporters		
(Álvarez-Sánchez et al. 2023)	*Rhipicephalus sanguineus* s.l	Cattle		x		CR		x		Transcriptomic analysis	Various
Amanzougaghene et al. (2018a) (Amanzougaghene et al. 2018b)	*Pediculus humanus humanus*	Humans		x		CR	x			Transcriptomic analysis	GluCl
Amanzougaghene et al. (2018b) (Amanzougaghene et al. 2018a)	*Pediculus humanus capitis*	Humans	x			SR	x			Genetic analysis	GluCl
(Buss et al. 2002)	*Culex pipiens* L. complex	NA			x	S			x	Lethal time bioassay	P-glycoprotein
Cafarchia et al. (2015) (Cafarchia et al. 2014)	*Rhipicephalus sanguineus* s.l	Dogs			x	S			x	Lethal time bioassay	ABC transporters
(Chaccour et al. 2017)	*Anopheles gambiae*	NA			x	S		x	x	Lethal time bioassay	CYP/p-glycoprotein
(Ferreira et al. 2023)	*Rhicephalus microplus*	Cattle	x			CR			x	Lethal time bioassay (with/without synergist)	ABC transporters
(Khangembam et al. 2018)	*Rhicephalus microplus*	Cattle	x			CR		x	x	Lethal time bioassay	P-glycoprotein/ABC transporter
Kim (2018) (Kim et al. 2018)	*Pediculus humanus humanus*	Humans			x	S		x	x	Liquid scintillation spectro-photometric analysis and transcriptomic analysis	ABC transporter and cytochrome P450
(Le Gall et al. 2018)	*Rhicephalus microplus*	Cattle	x			CR		x	x	Lethal time bioassay	Cytochrome P450, esterases, glutathione-S-transferase and ABC transporters
Mounsey (2007)	*Sarcoptes scabiei*	Humans	x			SR		x	x	Transcriptomic analysis	ABC transporters, Pgp, SsCl
(Mounsey et al. 2010)	*Sarcoptes scabiei*	Humans	x			SR		x		Transcriptomic analysis	Glutathione s-transferase
(Nicolas et al. 2021)	*Anopheles gambiae s.s*	NA			x	S		x		Lethal time bioassay (with/without synergist)	CYP/p-glycoprotein
(Pohl et al. 2011)	*Rhipicephalus microplus*	Cattle		x		CR			x	Lethal time bioassay	ABC transporter efflux pumps
(Pohl et al. 2014)	*Rhipicephalus microplus*	Cell line (originally collected from cattle)		x		CR			x	Transcriptomic analysis and bioassay	ABC transporter efflux pumps
(Ruiz-May et al. 2022)	*Rhicephalus microplus*	Cattle		x		CR		x	x	Transcriptomic analysis	Cytochrome P450, esterases, glutathione-S-transferase and ABC transporters
Shakya et al. (2022)	*Rhicephalus microplus*	Cattle	x		x	CR		x		Lethal time bioassay (with/without synergist)	Cytochrone-P450,glutathione-S-transferase and P-glycoprotein
(Yoon et al. 2011)	*Pediculus humanus*	Humans		x		DT			x	Transcriptomic analysis	ABC transporter genes

^*^FC field collected, *LR* lab-induced, *S* IVM-susceptible strain, *CYP* cytochrome, *GluCl* glutamate-gated chloride channels; **CR confirmed resistance based on laboratory testing, *SR* suspected resistance based on clinical observations, *S* susceptible, *DT* confirmed decreased tolerance based on laboratory testing; ***MRP multiresistant protein

### Target-site resistance

#### GluCl and complexin

Two of the 18 included studies assessed target-site resistance, while the remaining studies assessed mechanisms of metabolic resistance or changes to efflux pump expression (Amanzougaghene et al. 2018b; Amanzougaghene et al. 2018b). Both of these target-site resistance studies are related to ivermectin resistance in lice. In one study, headlice collected from individuals in Senegal who were experiencing treatment failure were genotyped and compared to a reference strain of body lice that were never exposed to ivermectin. The researchers identified three non-synonymous mutations in the GluCl gene region that only occurred in resistant headlice, suggestive of a potential role in ivermectin resistance (Amanzougaghene et al. 2018a). In a laboratory-based study by the same research group, body lice were reared on a rabbit model, and selected for resistance through exposure to ivermectin that was administered to the rabbit repeatedly, intravenously at sub-therapeutic doses. Transcriptional analysis of the resistant body lice suggested that complexin had an important role in resistance, as it was significantly upregulated in the resistant strain compared to a fully susceptible strain. However, no non-synonymous SNPs (single-nucleotide polymorphism), i.e. SNPs  that lead to a functional change, in the GluCl gene region were found to be associated with the resistant strain.

### Overexpression of multidrug resistance transporters

#### ABC transporters

Several studies have suggested that ivermectin resistance is associated with changes in ABC transporter gene expression, an efflux pump commonly associated with multi-resistance (Yoon et al. 2011; Ruiz-May et al. 2022; Gall et al. 2018; Khangembam et al. 2018; Kim et al. 2018; Buss et al. 2002; Pohl et al. 2014 Cafarchia et al. 2014; Pohl et al. 2011). A large study that analysed 12,712 field-collected tick samples and compared them to 6639 susceptible samples assessed the relationship between ivermectin resistance and the expression of several different detoxification genes (Gall et al. 2018). They found that ABC transporter gene expression was most strongly associated with ivermectin resistance, compared to other detoxification genes. Another, smaller scale study found similar results in bovine ticks that had been selected for ivermectin resistance (through exposure to sublethal doses of ivermectin) after analysing the ABC transporter transcriptome, where they also found that these genes were significantly upregulated (Pohl et al. 2014). The same research group established a resistant tick cell line by sequentially exposing cells to sub-lethal ivermectin concentrations. Again they found increased expression of ABC transporter genes, and increased susceptibility to ivermectin after the addition of cyclosporin A (Pohl et al. 2011), which is a known inhibitor of ABC transporters. A similar response was observed in the midgut of ticks and in body lice after exposure to ivermectin (Yoon et al. 2011; Ruiz-May et al. 2022).

Synergist bioassays on resistant and susceptible ectoparasites have also been conducted to assess the role of ABC transporters in ivermectin resistance. Two studies assessed the effect of an ABC transporter inhibitor (cyclosporin-A, MK571) in combination with ivermectin against field-collected ticks with confirmed resistance, and both found that it significantly enhanced the efficacy of ivermectin (Khangembam et al. 2018; Ferreira et al. 2023). They also showed that the addition of a P-glycoprotein inhibitor (verapamil) significantly reduced the LC_50_ (the concentration that is lethal to 50% of the individuals exposed) values of ivermectin. Another study in susceptible dog ticks showed that the addition of an ABC transporter inhibitor significantly increased the toxicity of ivermectin, further supporting evidence for the role of ABC transporter genes in ivermectin detoxification (Cafarchia et al. 2014).

Finally, a study investigating the role of ABC transporter C4 in ivermectin detoxification, Kim et al. used *X laevis* oocytes as an expression system to examine PhABCC4 expression, a gene which had previously shown to be significantly overexpressed in resistant body lice in Yoon et al. (Yoon et al. 2011; Kim et al. 2018). They demonstrated that PhABCC4 was directly involved with phase III metabolism of ivermectin. The addition of an ABC transporter inhibitor blocked metabolism of ivermectin by PhABCC4.

P-glycoproteins are specific ABC transporters, which pump molecules out of cells by an ATP-dependent mechanism. Buss et al. investigated the role of p-glycoprotein by expressing P-gp in cell culture, and through comparing *Culex* cell mortality with ivermectin with and without the addition of verapamil (Buss et al. 2002). They found that mortality was significantly higher when combined with verapamil. Shakya et al. also found that the addition of a P-glycoprotein inhibitor reduced LC_50_ levels in ticks, although this was not as significant as the addition of PBO (Shakya et al. 2022).

Multidrug resistance associated protein (MRP3) is a type of ABC transporter, structurally similar to P-glycoprotein. In one study that compared gene expression in ivermectin-exposed mites compared to unexposed mites, the authors found that MRP3 was significantly upregulated in ivermectin-exposed mites at all life stages (Mounsey 2007).

### Metabolic resistance

#### Cytochrome P450

Cytochrome (CYP) P450 has also been reported to have a role in ivermectin resistance in a number of studies (Ruiz-May et al. 2022; Gall et al. 2018; Kim et al. 2018; Shakya et al. 2022; Chaccour et al. 2017; Nicolas et al. 2021). Two transcriptomic analyses of ivermectin-resistant ticks compared to susceptible ticks found that cytochrome P450 had an important role in ivermectin resistance in ticks (Ruiz-May et al. 2022; Gall et al. 2018). Shakya et al. assessed the effect of adding synergists to resistant tick populations and found that there was a significant positive relationship between the resistance factor and the synergy factor with PBO and VER, suggesting that cytochrome P450 is involved in ivermectin resistance (Shakya et al. 2022). Kim et al. used baculovirus as expression systems for CYP6CJ1, which had previously shown to be significantly overexpressed in resistant body lice in Yoon et al. (Yoon et al. 2011; Kim et al. 2018). However, they did not find any evidence to confirm the role of CYP6CJ1 in ivermectin detoxification, although the authors suggest it may have an additional role in ivermectin sequestration, which refers to when drugs are restricted to certain tissues or compartments within the body.

A study in *Anopheles gambiae* mosquitoes feeding on a mini-pig model treated with ivermectin found that the addition of ketoconazole, an inhibitor of the CYP P450 and P-glycoprotein, resulted in increased mosquito mortality (Chaccour et al. 2017). This strengthens the evidence that suggest the role of these transporters and enzymes in ivermectin detoxification. Another study assessed the impact of CYP and P-glycoprotein inhibitors to investigate their respective roles in ivermectin metabolism within these mosquitoes (Nicolas et al. 2021). They investigated the effect of CYP and P-glycoprotein inhibitors on mosquito survival and concluded that simultaneous induction of both CYP and P-glycoprotein may be involved in ivermectin detoxification.

#### Esterases

Le Gall et al. assessed gene expression of various detoxification genes in resistant and susceptible ticks after the addition of different inhibitors and found that in ticks, esterase expression was strongly associated with ivermectin resistance (Gall et al. 2018).

#### Glutathione-S-transferase

A study of resistance mechanisms in scabies mites suggested that glutathione-S-transferase (GST) had a role in resistance after comparing its transcription levels in ivermectin-exposed mites with unexposed mites (Mounsey et al. 2010). Ruiz-May et al. also found overexpression of GST in ivermectin-resistant ticks (Ruiz-May et al. 2022). [46], corroborated this finding by showing that the addition of a GST inhibitor (diethyl maleate) led to reduced LC_50_ levels in resistant ticks (Shakya et al. 2022). Mounsey et al. found that GST2 was upregulated in ivermectin-exposed mites, compared to unexposed mites (Mounsey 2007). However, this difference was not statistically significant. No difference in GST1 expression was observed.

#### SsCl

One study investigated differences in the expression of and SsCl (a subtype of ion-gated channel) in mites after exposure to ivermectin. However, the authors observed no statistically significant difference (Mounsey 2007).

#### Other metabolism-related proteins

Alvaro-Sanchez et al. investigated the ovary proteome of IVM-resistant ticks compared to sensitive ticks in the ovary and found that there was a significant difference between 507 and 644 differentially expressed proteins (related to processes involved in detoxification), depending on the intensity of resistance (Álvarez-Sánchez et al. 2023).

## Discussion

Resistance mechanisms against existing insecticides used to target malaria vectors are fairly well understood. This knowledge has resulted in high quality, proactive monitoring of insecticide resistance, which has influenced control strategies, as well as the introduction of new tools such as dual-active ingredient interventions that inhibit detoxification enzymes associated with resistance to allow increased efficacy of insecticidal interventions (Protopopoff et al. 2018). A clearer understanding of ivermectin resistance mechanisms and the research gaps in this topic will help us prepare for both the monitoring of resistance in *Anopheles* mosquitoes, and the potential use of synergists in combination with ivermectin.

Considering the extensive use of ivermectin, to date, very little research has been conducted in this field, with only 18 studies identified in this review. The studies varied in terms of model organism, type of resistance, and methods used for assessment. Most studies, however, focused on species other than *Anopheles* mosquitoes, with the majority investigating ticks. The majority of studies focused on metabolic resistance and overexpression of multidrug resistance efflux pumps; there was very little investigation into target-site resistance, with only two studies that identified target-site resistance in lice. There was some consistency in the mechanisms of resistance identified, which may implicate similar resistance mechanisms in *Anopheles* mosquitoes, when they emerge.

A few studies directly assessed the role of various detoxification enzymes and efflux pumps in ivermectin metabolism and resistance. These findings describe or indicate several potential resistance mechanisms that have been studied in a number of different ectoparasites. There was substantial evidence supporting the role of ABC transporters and CYP in ivermectin resistance and detoxification. ABC transporter and CYP genes were upregulated in ivermectin resistant samples in a few of the included studies. The majority of these studies were in ticks; the studies conducted in lice and mosquitoes investigated susceptible or lab-induced resistant organisms. Beyond the ABC transporters and CYPs, there is also evidence supporting the role of GST and esterases in ivermectin resistance in ticks.

Regarding our understanding of target-site resistance mechanisms, there remains a significant gap in the evidence. Only two studies were identified, both of which investigated associations between genetic polymorphisms and ivermectin resistance in lice. They showed mixed results; one study suggested that resistant headlice collected from the field had genetic mutations in the GluCl gene. However, these findings were not supported by a follow-up study. The follow-up study did, however, suggest that mutations in the complexin gene region were significantly more common in resistant lice. Several of the possible molecular targets (genes of histamine-gated chloride channels, pH-gated chloride-channels, pyrantel and levamisole nicotinic acetylcholine receptors (nAChRs), and gamma amino butyric acid (GABA)-gated channels) that have been suggested as molecular targets of ivermectin have not been studied in ectoparasitic organisms.

The possible expansion in the use of high dose ivermectin for malaria would likely lead to increased selection pressure, and in order to mitigate the risk of ivermectin resistance developing, integrated resistance management strategies should be in place. The results of this scoping review identified several detoxification mechanisms that are implicated in ivermectin detoxification, as well as changes in efflux pump expression, which can be used to guide further research into the use of enzyme inhibitors in combination with ivermectin. In order to identify molecular markers of ivermectin resistance, further research into ivermectin target site resistance is needed.

Another important point of consideration, if ivermectin MDA is expanded, is the potential impact of ivermectin on resistance in non-target organisms. This issue has been largely neglected in existing vector control tools; however, given the direct effect and use of ivermectin on other parasites, it is essential that the impact of ivermectin MDA on the development of resistance in non-target organisms is monitored and mitigated (Huijben et al. 2023). One widely understood method of minimising the occurrence and spread of resistance is by limiting selection pressure. However, given that ivermectin has such a broad spectrum of uses, there will be limited control over the amount of selection pressure to which each organism is exposed to (Shoop 1993). The level of selection pressure an organism experiences under a given dosage and regimen depends on the discriminating dose it is associated with. In using ivermectin MDA in areas where multiple endoparasites and ectoparasites exist, the dosage and regimen that is chosen (likely the discriminating dose of the priority organism) will likely be used at the expense of other organisms and may result in a shorter time-to-emergence of resistance in the latter. Other factors that will influence this such as the genetic variability and reproduction rate of each organism must also be considered. In order to mitigate this effect on resistance in non-target organisms, ideally the highest safe dosage should be chosen.

The evidence summarised in this review is limited and indirectly addresses the review’s research question as many of the studies used artificially induced resistant or susceptible organisms, which may not reflect the mechanisms that develop in field settings. Differences in exposure to ivermectin, life history, and genetic diversity of targeted organisms in field settings may influence the mechanisms of resistance that develop. Furthermore, the organisms that were investigated in the studies we identified were mostly ticks; although we observed some consistency in terms of the mechanisms observed in different organisms, there were only two studies addressing resistance in *Anopheles* mosquitoes. It is not clear how much can be inferred from ivermectin resistance mechanisms from one organism to another.

Regardless of these limitations, the results of this review provide valuable insights regarding ivermectin mechanisms that have been identified in relevant ectoparasites in previous research and may inform future research and resistance monitoring strategies.

## Conclusion

- Several studies on ABC reporters and CYP have been conducted, and suggest that they are involved in ivermectin resistance and detoxification in several ectoparasitic organisms.
- A few studies have investigated the role of glutathione-S-transferase and esterases in ivermectin resistance in ticks and scabies mites.
- There is a large gap in the research related to target-site ivermectin resistance, with only two studies identified that investigated target-site resistance in lice.
- Close monitoring and further research of genetic changes associated with ivermectin should be incorporated into monitoring plans.

### Supplementary Information

Below is the link to the electronic supplementary material.

Supplementary file1 (DOCX 107 KB)

## Data Availability

Not applicable.
